# Supplementary material for: Effects of high-intensity interval training on selected indicators of physical fitness among male team-sport athletes: A systematic review and meta-analysis
Source: PLoS One. 2024 Nov 13;19(11):e0310955. doi: 10.1371/journal.pone.0310955 (PMC11559996; doi:10.1371/journal.pone.0310955)
Supplement: S1 Table — (DOCX) [file pone.0310955.s001.docx]

**Table S1. Detailed search strategy.**

**Searched on December 31, 2023**

| **Databases** | **Search strategy** | **Results** |
| --- | --- | --- |
| PubMed | ((((("high-intensity interval training"[Title/Abstract]) OR ("high-intensity intermittent training"[Title/Abstract])) OR ("HIIT"[Title/Abstract])) AND ("male team athletes"[Title/Abstract] OR "handball"[Title/Abstract] OR "basketball"[Title/Abstract] OR "soccer"[Title/Abstract] OR "football"[Title/Abstract])) AND ("physical performance"[Title/Abstract] OR "maximal oxygen uptake"[Title/Abstract] OR "VO2 max"[Title/Abstract] OR "aerobic capacity"[Title/Abstract] OR "repeated sprint ability"[Title/Abstract]) | 30 |
| Scopus | TITLE-ABS-KEY (“high-intensity interval training” OR “high-intensity intermittent training” OR “HIIT”) AND TITLE-ABS-KEY (“male team athletes” OR “handball” OR “basketball” OR “soccer” OR “football”) AND TITLE-ABS-KEY (“physical performance” OR “maximal oxygen uptake” OR “VO2 max” OR “aerobic capacity” OR “repeated sprint ability”) | 64 |
| Web of Science Core Collection | (((AB= ((“high-intensity interval training” OR “high-intensity intermittent training” OR “HIIT”))) AND AB= ((“male team athletes” OR “handball” OR “basketball” OR “soccer” OR “football”))) AND AB= (“physical performance” OR “maximal oxygen uptake” OR “VO2 max” OR “aerobic capacity” OR “repeated sprint ability”)) | 37 |
| SPORTDicus | AB (“high-intensity interval training” OR “high-intensity intermittent training” OR “HIIT”) AND AB (“male team athletes” OR “handball” OR “basketball” OR “soccer” OR “football”) AND AB (“physical performance” OR “maximal oxygen uptake” OR “VO2 max” OR “aerobic capacity” OR “repeated sprint ability”) | 9 |
| Total |  | 140 |
