# Supplementary material for: Effects of high-intensity interval training on selected indicators of physical fitness among male team-sport athletes: A systematic review and meta-analysis
Source: PLoS One. 2024 Nov 13;19(11):e0310955. doi: 10.1371/journal.pone.0310955 (PMC11559996; doi:10.1371/journal.pone.0310955)
Supplement: S3 Table — (DOCX) [file pone.0310955.s003.docx]

| **Table S3 The data used for meta-analysis** |  |  |  |  |  |  |  |  |  |  |  |  |
| --- | --- | --- | --- | --- | --- | --- | --- | --- | --- | --- | --- | --- |
| **Study** | **Sport** | **Test** | **HIIT pre-test** | | **HIIT post-test** | | **n** | **CG pre-test** | | **CG post-test** | | **n** |
|  |  |  | Mean | SD | Mean | SD |  | Mean | SD | Mean | SD |  |
| Arslan et al., 2022 [41] | Basketball | VO2max | 45.89 | 0.85 | 48.18 | 0.67 | 16 | 46.29 | 1.03 | 48.1 | 0.87 | 16 |
| Arslan et al., 2020 [42] | Soccer | VO2max | 46.8 | 0.6 | 48.9 | 0.9 | 10 | 47.2 | 1.3 | 48.8 | 0.8 | 10 |
| Boraczynski et al., 2023 [43] | Soccer | VO2max | 54.5 | 4.96 | 55.9 | 5.97 | 13 | 56.3 | 5.62 | 59.2 | 7.64 | 12 |
| Gantois et al., 2019 [46] | Basketball | VO2max | 49.24 | 5.5 | 50.25 | 4.62 | 9 | 48.38 | 4.34 | 46.43 | 2.62 | 8 |
| Kumari et al., 2023 [49] | Basketball | VO2max | 52.8 | 2.3 | 54.5 | 2.4 | 20 | 51.1 | 2.6 | 51.4 | 2.9 | 20 |
| Wells et al., 2014 [53] | Soccer | VO2max | 57.6 | 5.4 | 58.9 | 4.7 | 8 | 57.1 | 3.6 | 57.6 | 3.1 | 8 |
| Arslan et al., 2022 [41] | Basketball | YYIRT | 1130 | 100.66 | 1402.5 | 79.29 | 16 | 1177.5 | 122.17 | 1392.5 | 103.51 | 16 |
| Arslan et al., 2020 [42] | Soccer | YYIRT | 1240 | 75 | 1484 | 74 | 10 | 1284 | 152 | 1472 | 99 | 10 |
| Eniseler et al., 2017 [45] | Soccer | YYIRT | 2306.6 | 252.1 | 2480 | 158.7 | 9 | 2320 | 388 | 2432 | 336 | 10 |
| Kavaliauskas et al., 2017 [48] | Soccer | YYIRT | 1468 | 409 | 1643 | 382 | 7 | 1164 | 438 | 1171 | 433 | 7 |
| Lacono et al., 2015 [50] | Handball | YYIRT | 1297.8 | 300 | 1601.1 | 192 | 9 | 1364.4 | 397 | 1723.3 | 327 | 9 |
| Wells et al., 2014 [53] | Soccer | YYIRT | 896 | 37 | 987 | 44 | 8 | 891 | 46 | 888 | 42 | 8 |
| Arslan et al., 2022 [41] | Basketball | RSA_total_ | 36.66 | 0.83 | 34.62 | 0.79 | 16 | 36.87 | 1.29 | 35.03 | 1.3 | 16 |
| Arslan et al., 2020 [42] | Soccer | RSA_total_ | 38.2 | 1.7 | 34.9 | 1.5 | 10 | 37.8 | 1.5 | 35.6 | 1.2 | 10 |
| Gantois et al., 2019 [46] | Basketball | RSA_total_ | 29 | 2.3 | 28.07 | 1.28 | 9 | 29.08 | 1.56 | 29.27 | 1.34 | 8 |
| Hermassi et al., 2018 [47] | Handball | RSA_total_ | 37.6 | 0.66 | 36.6 | 0.7 | 15 | 37.5 | 1 | 37.6 | 0.96 | 15 |
| Salazar-Martinez et al., 2023 [52] | Soccer | RSA_total_ |  |  | 35.34 | 1.15 | 12 |  |  | 33.99 | 0.74 | 11 |
| Eniseler et al., 2017 RSAbest [45] | Soccer | RSA_best_ & RSA_mean_ | 6.75 | 0.19 | 6.81 | 0.18 | 9 | 6.73 | 0.19 | 6.96 | 0.24 | 10 |
| Eniseler et al., 2017 RSAmean [45] | Soccer | RSA_best_ & RSA_mean_ | 7.13 | 0.17 | 7.13 | 0.21 | 9 | 7.12 | 0.17 | 7.22 | 0.2 | 10 |
| Gantois et al., 2019 RSAbest [46] | Basketball | RSA_best_ & RSA_mean_ | 4.56 | 0.24 | 4.36 | 0.14 | 15 | 4.64 | 0.24 | 4.61 | 0.24 | 15 |
| Gantois et al., 2019 RSAmean [46] | Basketball | RSA_best_ & RSA_mean_ | 4.83 | 0.38 | 4.67 | 0.21 | 15 | 4.84 | 0.26 | 4.87 | 0.22 | 15 |
| Hermassi et al., 2018 RSAbest [47] | Handball | RSA_best_ & RSA_mean_ | 6.24 | 0.09 | 6.06 | 0.1 | 15 | 6.19 | 0.1 | 6.15 | 0.08 | 15 |
| Salazar-Martinez et al., 2023 RSAbest [52] | Soccer | RSA_best_ & RSA_mean_ | 4.25 | 0.11 | 4.2 | 0.11 | 12 | 4.19 | 0.1 | 4.09 | 0.09 | 11 |
| Salazar-Martinez et al., 2023 RSAmean [52] | Soccer | RSA_best_ & RSA_mean_ |  |  | 4.45 | 0.2 | 12 |  |  | 4.24 | 0.09 | 11 |

HIIT, high-intensity interval training; CG, control group, SD, standard deviation; YYIRT, Yo-Yo intermittent recovery training; RSA_total_, total sprint time; RSA_best_, best sprint time; RSA_mean_, average sprint time；In this study, if only pre-test data were missing and no significant differences were observed in the pre-test data for any variables (p > 0.05), the “Unmatched Groups” feature of the Meta-analysis software (CMA V3) was utilized to conduct statistical analysis on post-test data only.
