# Supplementary material for: Effects of high-intensity interval training on selected indicators of physical fitness among male team-sport athletes: A systematic review and meta-analysis
Source: PLoS One. 2024 Nov 13;19(11):e0310955. doi: 10.1371/journal.pone.0310955 (PMC11559996; doi:10.1371/journal.pone.0310955)
Supplement: S4 Table — (DOCX) [file pone.0310955.s004.docx]

**Data extraction table**

| **Study ID** | **Study Citation** | **Data Extractor & Data Extraction** | **Eligibility Confirmed (Yes/No)** | **Extracted Data/Variables** |
| --- | --- | --- | --- | --- |
| 1 | Arslan et al., 2022 [41]  International Journal of Environmental Research and Public Health | Yan Yandong  3/29/2024 | Yes | Sample size, Mean (SD)/  VO2max, YYIRT1, RSA_total_, VIFT |
| 2 | Arslan et al., 2020 [42]  Biology of Sport | Yan Yandong  3/29/2024 | Yes | Sample size, Mean (SD)/  VO2max, YYIRT1, RSA_total_ |
| 3 | Boraczyński et al., 2023 [43]  Journal of Sports Medicine and Physical Fitness | Yan Yandong  3/29/2024 | Yes | Sample size, Mean (SD)/  VO2max |
| 4 | Delextrat et al., 2018 [44]  Journal of Strength and Conditioning Research | Yan Yandong  3/29/2024 | Yes | Sample size, Mean (SD)/  VIFT |
| 5 | Eniseler et al., 2017 [45]  Journal of Human Kinetics | Yan Yandong  3/29/2024 | Yes | Sample size, Mean (SD)/  YYIRT1, RSA_best_ and RSA_mean_ |
| 6 | Gantois et al., 2019 [46]  Isokinetics and Exercise Science | Yan Yandong  3/29/2024 | Yes | Sample size, Mean (SD)/ VO2max, RSA_total_, RSA_best_ and RSA_mean_ |
| 7 | Hermassi et al., 2018 [47]  Isokinetics and Exercise Science | Yan Yandong  3/29/2024 | Yes | Sample size, Mean (SD)/  YYIRT1, RSA_total_, RSA_best_ |
| 8 | Kavaliauskas et al., 2017 [48]  The Journal of Sports Medicine and Physical Fitness | Yan Yandong  3/29/2024 | Yes | Sample size, Mean (SD)/  YYIRT1 |
| 9 | Kumari et al., 2023 [49]  Journal of Bodywork and Movement Therapies | Yan Yandong  3/29/2024 | Yes | Sample size, Mean (SD)/  VO2max |
| 10 | Lacono et al., 2015 [50]  Journal of Strength and Conditioning Research | Yan Yandong  3/29/2024 | Yes | Sample size, Mean (SD)/  YYIRT1 |
| 11 | Los Arcos et al., 2015 [51]  PLOS ONE | Yan Yandong  3/29/2024 | Yes | Sample size, Mean (SD)/  MAS |
| 12 | Salazar-Martínez et al., 2023 [52] Journal of Physical Education and Sport | Yan Yandong  3/29/2024 | Yes | Sample size, Mean (SD)/  RSA_total_, RSA_best_ and RSA_mean_ |
| 13 | Wells et al., 2014 [53]  Applied Physiology, Nutrition and Metabolism | Yan Yandong  3/29/2024 | Yes | Sample size, Mean (SD)/  VO2max, YYIRT2 |

**Notes:** VO2max, maximal oxygen uptake; YYIRT1 or 2, Yo-Yo Intermittent Recovery test level 1 or 2; RSA, repeated-sprint ability; VIFT, Velocity in 30–15 Intermittent Fitness Test; RSA_total_, total sprint time; RSA_best_, best sprint time; RSA_mean_, average sprint time.
