# Supplementary material for: Effects of high-intensity interval training on selected indicators of physical fitness among male team-sport athletes: A systematic review and meta-analysis
Source: PLoS One. 2024 Nov 13;19(11):e0310955. doi: 10.1371/journal.pone.0310955 (PMC11559996; doi:10.1371/journal.pone.0310955)
Supplement: Attachment — Submitted filename: Reviewer_PONE-D-24-16341.docx [file pone.0310955.s006.docx]

The research presented in this article is scientifically conceptualized and executed. However, some areas require attention to reach the level of publication.

The researchers have focussed on an important issue. Although they followed the necessary processes as a method, I must say that they could not put forward a structure that would reveal the quality of the study, especially in the introduction section.

In the introduction, starting with HIIT and continuing with team sports can be seen as a very sloppy behaviour for a systematic review and meta-analysis study. Therefore, my recommendation would be the most accurate reflection of HIIT training > HIIT training and soccer > in-depth physical and physiological effects of HIIT training and as the last paragraph, expressing the difference from other systematic review and meta-analysis studies that will explain the gap in the literature.
